# Two New PRP Conjugate Gradient Algorithms for Minimization Optimization Models

**DOI:** 10.1371/journal.pone.0140071

**Published:** 2015-10-26

**Authors:** Gonglin Yuan, Xiabin Duan, Wenjie Liu, Xiaoliang Wang, Zengru Cui, Zhou Sheng

**Affiliations:** 1 Guangxi Colleges and Universities Key Laboratory of Mathematics and Its Applications, College of Mathematics and Information Science, Guangxi University, Nanning, Guangxi, 530004, P. R. China; 2 School of Computer and Software, Nanjing University of Information Science & Technology, Nanjing 210044, P. R. China; 3 Jiangsu Engineering Center of Network Monitoring, Nanjing University of Information Science & Technology, Nanjing 210044, P. R. China; Nankai University, CHINA

## Abstract

Two new PRP conjugate Algorithms are proposed in this paper based on two modified PRP conjugate gradient methods: the first algorithm is proposed for solving unconstrained optimization problems, and the second algorithm is proposed for solving nonlinear equations. The first method contains two aspects of information: function value and gradient value. The two methods both possess some good properties, as follows: 1)*β*_*k*_ ≥ 0 2) the search direction has the trust region property without the use of any line search method 3) the search direction has sufficient descent property without the use of any line search method. Under some suitable conditions, we establish the global convergence of the two algorithms. We conduct numerical experiments to evaluate our algorithms. The numerical results indicate that the first algorithm is effective and competitive for solving unconstrained optimization problems and that the second algorithm is effective for solving large-scale nonlinear equations.

## Introduction

As we know, the conjugate gradient method is very popular and effective for solving the following unconstrained optimization problem

$$\begin{array}{c} \min_{x\in {ℜ}^{n}}f\left(x\right) \end{array}$$ (1)

where *f* : ℜ^*n*^ → ℜ is continuously differentiable and *g*(*x*) denotes the gradient of *f*(*x*) at *x*, the problem Eq (1) also can be applied to model some other problems [1–5]. The iterative formula used in the conjugate gradient method is usually given by

$$\begin{array}{c} x_{k+1}=x_{k}+\alpha_{k}d_{k} \end{array}$$ (2)

and

$$d_{k}=\{\begin{array}{cc} -g_{k} & \text{if}\;k=1\\ -g_{k}+\beta_{k}d_{k-1} & \text{if}\;k\geq 2 \end{array}$$ (3)

where *g*_*k*_ = *g*(*x*_*k*_), *β*_*k*_ ∈ ℜ is a scalar, *α*_*k*_ > 0 is a step length that is determined by some line search, and *d*_*k*_ denotes the search direction. Different conjugate methods have different choices for *β*_*k*_. Some of the popular methods [6–12] used to compute *β*_*k*_ are the DY conjugate gradient method [6], FR conjugate gradient method [7], PRP conjugate gradient method [8, 9], HS conjugate gradient method [10], LS conjugate gradient method [11], and CD conjugate gradient method [12]. *β*_*k*_ [8, 9] is defined by

$$\beta_{k}^{PRP}=\frac{g_{k}^{T}y_{k-1}}{∥g_{k-1}{∥}^{2}}$$ (4)

where ∥·∥ denotes the Euclidean norm, *y*_*k*−1_ = *g*_*k*_−*g*_*k*−1_. The PRP conjugate gradient method is currently considered to have the best numerical performance, but it does not have good convergence. With an exact line search, the global convergence of the PRP conjugate gradient method has been established by Polak and Ribière [8] for convex objective functions. However, Powell [13] proposed a counter example that proved the existence of nonconvex functions on which the PRP conjugate gradient method does not have global convergence, even with an exact line search. With the weak Wolfe-Powell line search, Gilbert and Nocedal [14] proposed a modified PRP conjugate gradient method by restricting *β*_*k*_ to be not less than zero and proved that it has global convergence, with the hypothesis that it satisfies the sufficient descent condition. Gilbert and Nocedal [14] also gave an example showing that *β*_*k*_ may be negative even though the objective function is uniformly convex. When the Strong Wolfe-Powell line search was used, Dai [15] gave a example showing that the PRP method cannot guarantee that every step search direction is the descent direction, even if the objective function is uniformly convex.

Through the above observations and [13, 14, 16–18], we know that the following sufficient descent condition

$$\begin{array}{c} -g_{k}^{T}d_{k}\geq b∥g_{k}{∥}^{2},\;\forall b>0 \end{array}$$ (5)

and the condition *β*_*k*_ is not less than zero are very important for establishing the global convergence of the conjugate gradient method.

The weak Wolfe-Powell (WWP) line search is designed to compute *α*_*k*_ and is usually used for the global convergence analysis. The WWP line search is as follows

$$\begin{array}{c} f\left(x_{k}+\alpha_{k}d_{k}\right)\leq f\left(x_{k}\right)+\delta_{1}\alpha_{k}g_{k}^{T}d_{k} \end{array}$$ (6)

and

$$\begin{array}{c} g{\left(x_{k}+\alpha_{k}d_{k}\right)}^{T}d_{k}\geq \delta_{2}g_{k}^{T}d_{k} \end{array}$$ (7)

where $\delta_{1}\in \left(0,\;\frac{1}{2}\right),\;\delta_{2}\in \left(\delta_{1},\;1\right)$.

Recently, many new conjugate gradient methods ([19–28] etc.) that possess some good properties have been proposed for solving unconstrained optimization problems.

In Section 2, we state the motivation behind our approach and give a new modified PRP conjugate gradient method and new algorithm for solving problem Eq (1). In Section 3, we prove that the search direction of our new algorithm satisfies the sufficient descent property and trust region property; moreover, we establish the global convergence of the new algorithm with the WWP line search. In Section 4, we provide numerical experiment results for some test problems.

## New algorithm for unconstrained optimization

Wei et al. [29] give a new PRP conjugate gradient method usually called the WYL method. When the WWP line search is used, this WYL method has global convergence under the sufficient descent condition. Zhang [30] give a modified WYL method called the NPRP method as follows

$$\beta_{k}^{NPRP}=\frac{{\left\|g_{k}\right\|}^{2}-\frac{∥g_{k}∥}{∥g_{k-1}∥}\;\left|g_{k}^{T}g_{k-1}\right|}{∥g_{k-1}{∥}^{2}}$$

The NPRP method possesses better convergence properties. The above formula for *y*_*k*−1_ contains only gradient value information, but some new *y*_*k*−1_ formulas [31, 32] contain information on gradient value and function value. Yuan et al.[32] propose a new *y*_*k*−1_ formula as follows

$$y_{k-1}^{m}=y_{k-1}+\frac{\max\{\rho_{k-1},0\}}{{∥s_{k-1}∥}^{2}}s_{k-1},$$

and

$$\rho_{k-1}=2\;\left[f\left(x_{k-1}\right)-f\left(x_{k}\right)\right]+{\left(g\left(x_{k}\right)+g\left(x_{k-1}\right)\right)}^{T}s_{k-1}.$$

Where *s*_*k*−1_ = *x*_*k*_−*x*_*k*−1_.

Li and Qu [33] give a modified PRP conjugate method as follows

$$\beta_{k}=\frac{g_{k}^{T}y_{k-1}}{max\{\;t∥d_{k-1}∥,\;{∥g_{k-1}∥}^{2}\}}\;,\;\;t>0$$

and

$$d_{k}=-g_{k}-\beta_{k}\frac{g_{k}^{T}d_{k-1}}{{∥g_{k}∥}^{2}}g_{k}+\beta_{k}d_{k-1},\;\;d_{0}=-g_{0}.$$

Under suitable conditions, Li and Qu [33] prove that the modified PRP conjugate method has global convergence.

Motivated by the above discussions, we propose a new modified PRP conjugate method as follows

$$\beta_{k}^{BPRP}=\frac{\text{min}\left\{\left|g_{k}^{T}y_{k-1}^{m}\right|\;,\;u_{1}\left({\left\|g_{k}\right\|}^{2}-\frac{\left\|g_{k}\right\|}{\left\|g_{k-1}\right\|}\left|g_{k}^{T}g_{k-1}\right|\right)\right\}}{u_{2}\left\|d_{k-1}\right\|\;\left\|y_{k-1}\right\|+{\left\|g_{k-1}\right\|}^{2}}$$ (8)

and

$$\begin{array}{c} d_{k}=\{\begin{array}{cc} -g_{k} & \text{if}\;k=1\\ -g_{k}-\beta_{k}^{BPRP}\frac{g_{k}^{T}d_{k-1}}{∥g_{k}{∥}^{2}}g_{k}+\beta_{k}^{BPRP}d_{k-1} & \text{if}\;k\geq 2 \end{array} \end{array}$$ (9)

where *u*_1_ > 0, *u*_2_ > 0, $y_{k-1}^{m}$ is the $y_{k-1}^{m}$ of [32].

**Fig 1:**
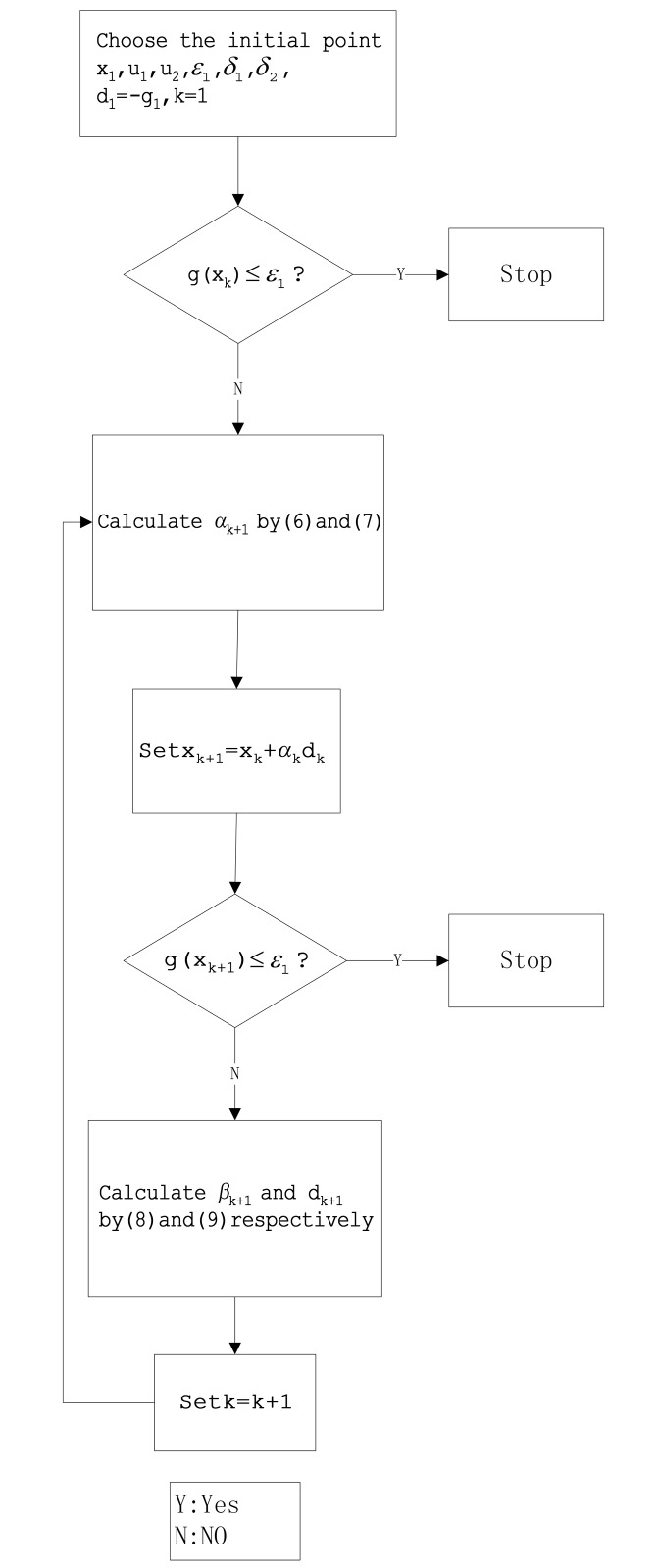
The diagram about Algorithm 2.1.

As $∥g_{k}{∥}^{2}-\frac{∥g_{k}∥}{∥g_{k-1}∥}\left|g_{k}^{T}g_{k-1}\right|\geq 0,$ it follows directly from the above formula that $\beta_{k}^{BPRP}\geq 0$. Next, we present a new algorithm and it’s diagram (Fig 1) as follows.

**Algorithm 2.1**

**Step 0:** Given the initial point $x_{1}\in {ℜ}^{n},\;u_{1}>0,\;u_{2}>0,\varepsilon_{1}\geq 0,0<\delta_{1}<\frac{1}{2},\delta_{1}<\delta_{2}<1$, set *d*_1_ = −∇*f*(*x*_1_) = −*g*_1_, *k*: = 1.

**Step 1:** Calculate $∥g_{k}∥$; if $∥g_{k}∥\leq \varepsilon_{1}$, stop; otherwise, go to step 2.

**Step 2:** Calculate step length *α*_*k*_ by the WWP line search.

**Step 3:** Set *x*_*k*+1_ = *x*_*k*_ + *α*_*k*_ *d*_*k*_, then calculate $∥g_{k+1}∥$; if $∥g_{k+1}∥\leq \varepsilon_{1}$, stop; otherwise, go to step 4.

**Step 4:** Calculate the scalar *β*_*k*+1_ by Eq (8) and calculate the search direction *d*_*k*+1_ by Eq (9).

**Step 5:** Set *k*: = *k* + 1; go to step 2.

## Global convergence analysis

Some suitable assumptions are often used to analyze the global convergence of the conjugate gradient method. Here, we state it as follows

**Assumption 3.1**

1. The level set Ω = {*x* ∈ ℜ^*n*^ ∣ *f*(*x*) ≤ *f*(*x*_1_)} is bounded.
2. In some neighborhood *H* of Ω, *f* is a continuously differentiable function, and the gradient function *g* of *f* is Lipschitz continuous, namely, there exists a constant *L* > 0 such that
  ‖g(x)−g(y)‖≤L ‖x−y‖, ∀x,y∈H (10)

By Assumption 3.1, it is easy to obtain that there exist two constants *A* > 0 and *η*_1_ > 0 satisfying

$$∥x∥\;\leq A,\;∥g\left(x\right)∥\;\leq \eta_{1},\;\forall x\in \Omega$$ (11)

**Lemma 0.1** *Let the sequence* {*d*_*k*_} *be generated by* Eq (9); *then, we have*

$$g_{k}^{T}d_{k}=-{\left\|g_{k}\right\|}^{2},\;\forall k\geq 1$$ (12)

**Proof** When *k* = 1, we can obtain $g_{1}^{T}d_{1}=-\;\|g_{1}{\|}^{2}$ by Eq (9), so Eq (12) holds. When *k* ≥ 2, we can obtain

$$\begin{array}{l} g_{k}^{T}d_{k}\;=\;g_{k}^{T}\left(-g_{k}-\beta_{k}^{BPRP}\frac{g_{k}^{T}d_{k-1}}{∥g_{k}{∥}^{2}}g_{k}+\beta_{k}^{BPRP}d_{k-1}\right)\\ \;=\;-∥g_{k}{∥}^{2} \end{array}$$

The proof is achieved.

We know directly from above Lemma that our new method has the sufficient descent property.

**Lemma 0.2** *Let the sequence* {*x*_*k*_} *and* {*d*_*k*_, *g*_*k*_} *be generated by Algorithm 2.1, and suppose that Assumption 3.1 holds; then, we can obtain*

$$\begin{array}{c} \sum_{k=1}^{\infty }\frac{{\left(g_{k}^{T}d_{k}\right)}^{2}}{{∥d_{k}∥}^{2}}<+\infty \end{array}$$ (13)

**Proof** By Eq (7) and the Cauchy-Schwarz inequality, we have

$$-\left(1-\delta_{2}\right)g_{k}^{T}d_{k}\leq \;∥g_{k+1}-g_{k}∥\;∥d_{k}∥$$

Combining the above inequality with Assumption 3.1 ii) generates

$$-\left(1-\delta_{2}\right)g_{k}^{T}d_{k}\leq L\alpha_{k}∥d_{k}{∥}^{2}$$

it is easy to know $g_{k}^{T}d_{k}\leq 0$ by lemma 0.1. By combining the above inequality with Eq (6), we obtain

$$f_{k}-f_{k+1}\geq \frac{\delta_{1}\left(1-\delta_{2}\right)}{L}\frac{{\left(g_{k}^{T}d_{k}\right)}^{2}}{{∥d_{k}∥}^{2}}.$$

Summing up the above inequalities from *k* = 1 to *k* = ∞, we can deduce that

$$\frac{\delta_{1}\left(1-\delta_{2}\right)}{L}\sum_{k=1}^{\infty }\frac{{\left(g_{k}^{T}d_{k}\right)}^{2}}{{∥d_{k}∥}^{2}}\leq f_{1}-f_{\infty }.$$

By Eq (6), Assumption 3.1 and lemma 0.1, we know that {*f*_*k*_} is bounded below, so we obtain

$$\sum_{k=1}^{\infty }\frac{{\left(g_{k}^{T}d_{k}\right)}^{2}}{{∥d_{k}∥}^{2}}<+\infty .$$

This finishes the proof.

The Eq (13) is usually called the Zoutendijk condition [34], and it is very important for establishing global convergence.

**Lemma 0.3** *Let the sequence* {*β*_*k*_, *d*_*k*_} *be generated by Algorithm 2.1, we have*

$$\begin{array}{c} ∥d_{k}∥\leq N∥g_{k}∥ \end{array}$$ (14)

*where* $N=1+\frac{4u_{1}}{u_{2}}$.

**Proof** When *d*_*k*_ = 0, we directly get *g*_*k*_ = 0 from Eq (12). When *d*_*k*_ ≠ 0, by the Cauchy-Schwarz inequality, we can easily obtain

$$∥g_{k}{∥}^{2}-\frac{∥g_{k}∥}{∥g_{k-1}∥}\left|g_{k}^{T}g_{k-1}\right|\leq g_{k}^{T}\left(g_{k}-\frac{∥g_{k}∥}{∥g_{k-1}∥}g_{k-1}\right)$$

and

$$\begin{array}{ccc} g_{k}^{T}\left(g_{k}-\frac{∥g_{k}∥}{∥g_{k-1}∥}g_{k-1}\right) & \leq & ∥g_{k}∥\;\left\|\left(g_{k}-g_{k-1}\right)+\left(g_{k-1}-\frac{∥g_{k}∥}{∥g_{k-1}∥}g_{k-1}\right)\right\|\\ & \leq & 2∥g_{k}∥\;∥g_{k}-g_{k-1}∥ \end{array}$$

We can obtain

$$∥g_{k}{∥}^{2}-\frac{∥g_{k}∥}{∥g_{k-1}∥}\;\left|g_{k}^{T}g_{k-1}\right|\;\leq 2\;∥g_{k}∥\;∥y_{k-1}∥$$

Using Eq (8), we have

$$\begin{array}{ccc} \left|\beta_{k}^{BPRP}\right| & \leq & \frac{u_{1}\left(∥g_{k}{∥}^{2}-\frac{∥g_{k}∥}{∥g_{k-1}∥}\;\left|g_{k}^{T}g_{k-1}\right|\right)}{u_{2}∥d_{k-1}∥\;∥y_{k-1}∥}\\ & \leq & \frac{2u_{1}}{u_{2}}\frac{∥g_{k}∥}{∥d_{k-1}∥} \end{array}$$

Finally, when *k* ≥ 2 by Eq (9), we have

$$\begin{array}{ccc} ∥d_{k}∥ & \leq & \left\|g_{k}\right\|+\left|\beta_{k}^{BPRP}\left|\frac{∥g_{k}∥∥d_{k-1}∥}{∥g_{k}{∥}^{2}}∥g_{k}∥+\right|\beta_{k}^{BPRP}\right|\;\left\|d_{k-1}\right\|\\ & \leq & \left\|g_{k}\right\|+\frac{2u_{1}}{u_{2}}∥g_{k}∥+\frac{2u_{1}}{u_{2}}\left\|g_{k}\right\|\\ & \leq & \left(1+\frac{4u_{1}}{u_{2}}\right)\left\|g_{k}\right\| \end{array}$$

Let $N=1+\frac{4u_{1}}{u_{2}}$; we obtain $∥d_{k}∥\leq N∥g_{k}∥$. This finishes the proof.

This lemma also shows that the search direction of our algorithm has the trust region property.

**Theorem 0.1** *Let the sequence* {*d*_*k*_, *g*_*k*_, *β*_*k*_} *and* {*x*_*k*_} *be generated by Algorithm 2.1. Suppose that Assumption 3.1 holds; then*

$$\begin{array}{c} \lim_{k\to \infty }\;∥g_{k}∥=0 \end{array}$$ (15)

**Proof** By Eqs (12) and (13), we obtain

$$\begin{array}{c} \sum_{k=1}^{\infty }\frac{{∥g_{k}∥}^{4}}{{∥d_{k}∥}^{2}}<+\infty \end{array}$$ (16)

By Eq (14), we have ${\left\|d_{k}\right\|}^{2}\leq N^{2}{\left\|g_{k}\right\|}^{2}$; then, we obtain

$${\left\|g_{k}\right\|}^{2}\leq N^{2}\frac{{∥g_{k}∥}^{4}}{{∥d_{k}∥}^{2}},$$

which together with Eq (16) can yield

$$\sum_{k=1}^{\infty }{∥g_{k}∥}^{2}\leq N^{2}\sum_{k=1}^{\infty }\frac{{∥g_{k}∥}^{4}}{{∥d_{k}∥}^{2}}<+\infty .$$

From the above inequality, we can obtain $\lim_{k\Rightarrow \infty }\;∥g_{k}∥=0$. The proof is finished.

## Numerical Results

When *β*_*k*+1_ and *d*_*k*+1_ are calculated by Eqs (4) and (3), respectively, in step 4 of Algorithm 2.1, we call it the PRP conjugate gradient algorithm. We test Algorithm 2.1 and the PRP conjugate gradient algorithm using some benchmark problems. The test environment is MATLAB 7.0, on a Windows 7 system. The initial parameters are given by

$$u_{1}=1,\;u_{2}=2,\delta_{1}=0.2,\;\delta_{2}=0.8,\;\varepsilon_{1}=10^{-6}.$$

We use the following Himmeblau stop rule, which satisfies

If ∣*f*(*x*_*k*_)∣ ≤ *ɛ*_2_, let *stop*1 = $stop1=\;\left|f\left(x_{k}\right)-f\left(x_{k+1}\right)\right|$; otherwise, let $stop1=\frac{\left|f\left(x_{k}\right)-f\left(x_{k+1}\right)\right|}{\left|f\left(x_{k}\right)\right|}$. The test program will be stopped if *stop*1 < *ɛ*_3_ or $∥g(x_{k})∥<\varepsilon_{1}$ is satisfied, where *ɛ*_2_ = *ɛ*_3_ = 10^−6^. When the total number of iterations is greater than one thousand, the test program will be stopped. The test results are given in Tables 1 and 2: *x*_1_ denotes the initial point, Dim denotes the dimension of test function, NI denotes the the total number of iterations, and NFG = NF+NG (NF and NG denote the number of the function evaluations and the number of the gradient evaluations, respectively). $f^{{}^{\prime }}$ denotes the function value when the program is stopped. The test problems are defined as follows.

1. Schwefel function:
  $$f_{Sch}\left(x\right)=418.9829n+\sum_{i=1}^{n}x_{i}\sin\sqrt{\left|x_{i}\right|},x_{i}\in \left[-512.03,\;511.97\right],$$
  $$x^{*}=\left(-420.9687,-420.9687,...,-420.9687\right),f_{Sch}\left(x^{*}\right)=0.$$
2. Langerman function:
  $$f_{Lan}\left(x\right)=-\sum_{i=1}^{m}c_{i}e^{-\frac{1}{\pi }\sum_{j=1}^{n}{\left(x_{j}-a_{ij}\right)}^{2}}\cos\left(\pi \sum_{j=1}^{n}{\left(x_{j}-a_{ij}\right)}^{2}\right),\;x_{i}\in \left[0,\;10\right],\;m=n,\;$$
  $$x^{*}=random,\;f_{Lan}\left(x^{*}\right)=random.$$
3. Schwefel′s function
  $$f_{SchDS}\left(x\right)=\overset{n}{\underset{i=1}{\sum }}{\left(\overset{i}{\underset{j=1}{\sum }}x_{j}\right)}^{2},\;x_{i}\in \left[-65.536,\;65.536\right],\;$$
  $$x^{*}=\left(0,0,...,0\right),\;f_{SchDS}\left(x^{*}\right)=0.$$
4. Sphere function:
  $$f_{Sph}\left(x\right)=\sum_{i=1}^{n}x_{i}^{2},\;\;x_{i}\in \left[-5.12,\;5.12\right],\;\;$$
  $$x^{*}=\left(0,0,\;.\;.\;.\;,\;0\right),\;\;f_{Sph}\left(x^{*}\right)=0.$$
5. Griewangk function:
  $$f_{Gri}\left(x\right)=1+\sum_{i=1}^{n}\frac{x_{i}^{2}}{4000}-\prod_{i=1}^{n}\cos\;(\frac{x_{i}}{\sqrt{i}}),\;x_{i}\in \left[-600,\;600\right],\;$$
  $$x^{*}=\left(0,\;0,\;.\;.\;.\;,\;0\right),\;f_{Gri}\left(x^{*}\right)=0.$$
6. Rosenbrock function:
  $$f_{Ros}\left(x\right)=\sum_{i=1}^{n-1}[100{\left(x_{i+1}-x_{i}^{2}\right)}^{2}+{\left(x_{i}-1\right)}^{2}],\;x_{i}\in \left[-2.048,\;2.048\right],\;$$
  $$x^{*}=\left(1,\;...,\;1\right),\;f_{Ros}\left(x^{*}\right)=0.$$
7. Ackley function:
  $$f_{Ack}\left(x\right)=20+e-20e^{-0.2\sqrt{\frac{1}{n}\sum_{i=1}^{n}x_{i}^{2}}\;}-e^{\frac{1}{n}\sum_{i=1}^{n}\cos\;(2\pi x_{i})},\;x_{i}\in \left[-30,\;30\right],\;$$
  $$x^{*}=\left(0,\;0,\;.\;.\;.\;,0\right),\;f_{Ack}\left(x^{*}\right)=0.$$
8. Rastrigin function:
  $$f_{Ras}\left(x\right)=10n+\sum_{i=1}^{n}\left(x_{i}^{2}-10\cos\left(2\pi x_{i}\right)\right),\;x_{i}\in \left[-5.12,\;5.12\right],\;$$
  $$x^{*}=\left(0,0,...,0\right),\;f_{Ras}\left(x^{*}\right)=0.$$

**Table 1:** Test results for Algorithm 2.1.

Problems	Dim	*x*_1_	NI/NFG	*f′*
1	50	(-426,-426,…,-426)	2/9	6.363783e-004
	120	(-426,-426,…,-426)	2/9	1.527308e-003
	200	(-426,-426,…,-426)	2/9	2.545514e-003
	1000	(-410,-410,…,-410)	3/12	1.272757e-002
2	50	(3,3,…,3)	0/2	-1.520789e-060
	120	(5,5,…,5)	0/2	0.000000e+000
	200	(6,6,…,6)	0/2	0.000000e+000
	1000	(1,1,…,1)	0/2	-7.907025e-136
3	50	(-0.00001,0,-0.00001,0,…)	2/8	1.561447e-009
	120	(-0.00001,0,-0.00001,0,…)	2/8	1.769900e-008
	200	(-0.00001,0,-0.00001,0,…)	2/8	7.906818e-008
	1000	(0.000001,0,0.000001,0,…)	2/8	9.619586e-008
4	50	(-4,-4,…,-4)	1/6	1.577722e-028
	120	(-2,-2,…,-2)	1/6	3.786532e-028
	200	(1,1,…,1)	1/6	7.730837e-027
	1000	(3,3,…,3)	1/6	1.079951e-024
5	50	(-7,0,-7,0,…)	2/10	0.000000e+000
	120	(0.592,0,0.592,0,…)	4/14	3.183458e-007
	200	(0.451,0,0.451,0,…)	4/14	3.476453e-007
	1000	(0.38,0,0.38,0,…)	1/6	0.000000e+000
6	50	(1.001,1.001,…,1.001)	2/36	4.925508e-003
	120	(1.001,1.001,…,1.001)	2/36	1.198551e-002
	200	(1.001,1.001,…,1.001)	2/36	2.006158e-002
	1000	(1.001,1.001,…,1.001)	2/36	1.009107e-001
7	50	(0.01,0,0.01,0,…)	0/2	3.094491e-002
	120	(-0.05,0,-0.05,0,…)	0/2	2.066363e-001
	200	(0.01,0,0.01,0,…)	0/2	3.094491e-002
	1000	(0.07,0,0.07,0,…)	0/2	3.233371e-001
8	50	(0.003,0.003,…,0.003)	3/26	0.000000e+000
	120	(0.005,0.005,…,0.005)	2/9	0.000000e+000
	200	(0.006,0,0.006,0,…)	2/9	0.000000e+000
	1000	(0.015,0.015,…,0.015)	2/8	0.000000e+000

**Table 2:** Test results for the PRP conjugate gradient algorithm.

Problems	Dim	*x*_1_	NI/NFG	*f′*
1	50	(-426,-426,…,-426)	2/24	6.363783e-004
	120	(-426,-426,…,-426)	2/11	1.527308e-003
	200	(-426,-426,…,-426)	3/41	2.545514e-003
	1000	(-410,-410,…,-410)	3/41	1.272757e-002
2	50	(3,3,…,3)	0/2	-1.520789e-060
	120	(5,5,…,5)	0/2	0.000000e+000
	200	(6,6,…,6)	0/2	0.000000e+000
	1000	(1,1,…,1)	0/2	-7.907025e-136
3	50	(-0.00001,0,-0.00001,0,…)	2/8	1.516186e-009
	120	(-0.00001,0,-0.00001,0,…)	2/8	1.701075e-008
	200	(-0.00001,0,-0.00001,0,…)	2/8	7.579825e-008
	1000	(0.000001,0,0.000001,0,…)	2/8	9.198262e-008
4	50	(-4,-4,…,-4)	1/6	1.577722e-028
	120	(-2,-2,…,-2)	1/6	3.786532e-028
	200	(1,1,…,1)	1/6	7.730837e-027
	1000	(3,3,…,3)	1/6	1.079951e-024
5	50	(-7,0,-7,0,…)	4/16	3.597123e-013
	120	(0.592,0,0.592,0,…)	5/17	3.401145e-007
	200	(0.451,0,0.451,0,…)	5/17	4.566281e-007
	1000	(0.38,0,0.38,0,…)	1/6	0.000000e+000
6	50	(1.001,1.001,…,1.001)	2/36	4.925508e-003
	120	(1.001,1.001,…,1.001)	2/36	1.198551e-002
	200	(1.001,1.001,…,1.001)	2/36	2.006158e-002
	1000	(1.001,1.001,…,1.001)	2/36	1.009107e-001
7	50	(0.01,0,0.01,0,…)	0/2	3.094491e-002
	120	(-0.05,0,-0.05,0,…)	0/2	2.066363e-001
	200	(0.01,0,0.01,0,…)	0/2	3.094491e-002
	1000	(0.07,0,0.07,0,…)	0/2	3.233371e-001
8	50	(0.003,0.003,…,0.003)	2/10	0.000000e+000
	120	(0.005,0.005,…,0.005)	2/10	0.000000e+000
	200	(0.006,0,0.006,0,…)	2/10	0.000000e+000
	1000	(0.015,0.015,…,0.015)	2/22	3.636160e-009

It is easy to see that the two algorithms are effective for the above eight test problems listed in Tables 1 and 2. We use the tool of Dolan and Morè [35] to analyze the numerical performance of the two algorithms.

**Fig 2:**
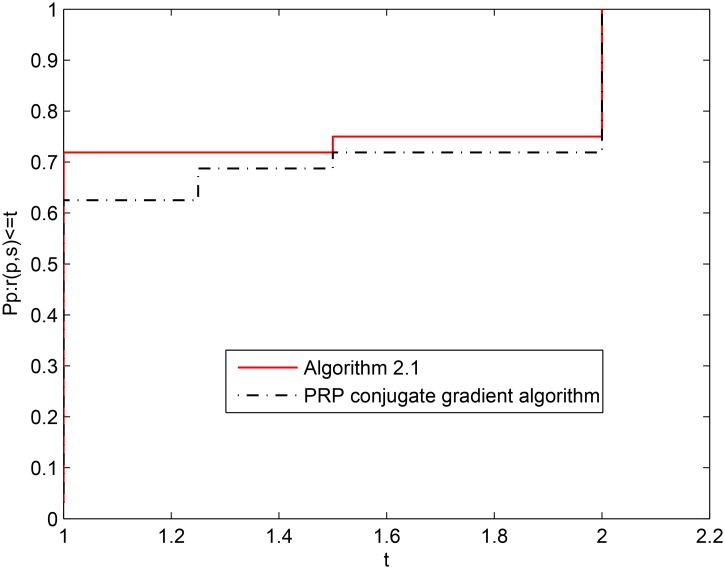
Performance profiles of the two algorithms (NI).

**Fig 3:**
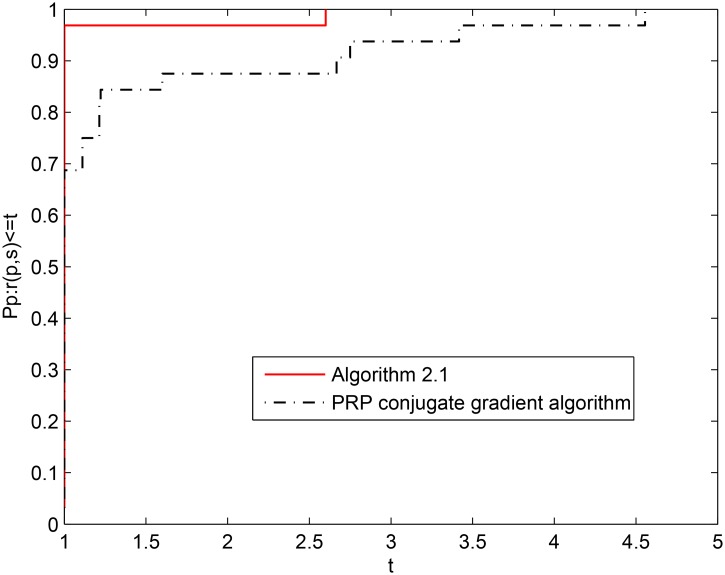
Performance profiles of the two algorithms (NFG).

For the above eight test problems, Fig 2 shows the numerical performance of the two algorithms when the information of NI is considered, and Fig 3 shows the the numerical performance of the two algorithms when the information of NFG is considered. From the above two figures, it is easy to see that Algorithm 2.1 yields a better numerical performance than the PRP conjugate gradient algorithm on the whole. From Tables 1 and 2 and the two figures, we can conclude that Algorithm 2.1 is effective and competitive for solving unconstrained optimization problems.

A new algorithm is given for solving nonlinear equations in the next section. The sufficient descent property and the trust region property of the new algorithm are proved in Section 6; moreover, we establish the global convergence of the new algorithm. In Section 7, the numerical results are presented.

## New algorithm for nonlinear equations

We consider the system of nonlinear equations

$$\begin{array}{c} q\left(x\right)=0,\;x\in {ℜ}^{n}. \end{array}$$ (17)

where *q* : ℜ^*n*^ → ℜ^*n*^ is a continuously differentiable and monotonic function. ∇*q*(*x*) denotes the Jacobian matrix of *q*(*x*); if ∇*q*(*x*) is symmetric, we call Eq (17) symmetric nonlinear equations. As *q*(*x*) is monotonic, the following inequality

$${\left(q\left(x\right)-q\left(y\right)\right)}^{T}\left(x-y\right)\geq 0,\;\forall x,\;y\in {ℜ}^{n}$$

holds. If a norm function is defined as follows

$$h\left(x\right)=\frac{1}{2}\;{\left\|q\left(x\right)\right\|}^{2}$$

and we define the unconstrained optimization problem as follows,

$$\begin{array}{c} \min h\left(x\right),\;x\in {ℜ}^{n} \end{array}$$ (18)

We know directly that the problem Eq (17) is equivalent to the problem Eq (18).

The iterative formula Eq (2) is also usually used in many algorithms for solving problem Eq (17). Many algorithms ([36–41], etc.) have been proposed for solving special classes of nonlinear equations. We are more interested in the process of dealing with large-scale nonlinear equations. By Eq (2), it is easy to see that the two factors of stepsize *α*_*k*_ and search direction *d*_*k*_ are very important for dealing with large-scale problems. When dealing with large-scale nonlinear equations and unconstrained optimization problems, there are many popular methods ([38, 42–46] etc.) for computing *d*_*k*_, such as conjugate gradient methods, spectral gradient methods, and limited-memory quasi-Newton approaches. Some new line search methods [37, 47] have been proposed for calculating *α*_*k*_. Li and Li [48] provide the following new derivative-free line search method

$$\begin{array}{c} -q{\left(x_{k}+\alpha_{k}d_{k}\right)}^{T}d_{k}\geq \sigma_{3}\alpha_{k}\left\|q\left(x_{k}+\alpha_{k}d_{k}\right)\right\|\;{\left\|d_{k}\right\|}^{2}, \end{array}$$ (19)

where *α*_*k*_ = max{*γ*, *ργ*, *ρ* ^2^ *γ*, …}, *ρ* ∈ (0,1), *σ*_3_ > 0 and *γ* > 0. This line search method is very effective for solving large-scale nonlinear monotonic equations.

Solodov and Svaiter [49] presented a hybrid projection-proximal point algorithm that could conquer some drawbacks when the form Eq (18) is used with nonlinear equations. Yuan et al.[50] proposed a three-term PRP conjugate gradient algorithm by using the projection-based technique, which was introduced by Solodov et al.[51] for optimization problems. The projection-based technique is very effective for solving nonlinear equations. It involves certain methods to compute search direction *d*_*k*_ and certain line search methods to calculate *α*_*k*_, which satisfies

$$q{\left(w_{k}\right)}^{T}\left(x_{k}-w_{k}\right)>0$$

in which *w*_*k*_ = *x*_*k*_ + *α*_*k*_ *d*_*k*_. For any x* that satisfies *q*(x*) = 0, considering that *q*(*x*) is monotonic, we can obtain

$$q{\left(w_{k}\right)}^{T}\left(x^{*}-w_{k}\right)\leq 0.$$

Thus, it is easy to obtain the current iterate *x*_*k*_, which is strictly separated from the zeros of the system of equations Eq (17) by the following hyperplane

$$T_{k}=\{x\in {ℜ}^{n}|q{\left(w_{k}\right)}^{T}\left(x-w_{k}\right)=0\}$$

Then, the iterate *x*_*k*+1_ can be obtained by projecting *x*_*k*_ onto the above hyperplane. The projection formula can be set as follows

$$\begin{array}{c} x_{k+1}=x_{k}-\frac{q{\left(w_{k}\right)}^{T}\left(x_{k}-w_{k}\right)}{{∥q(w_{k})∥}^{2}}q\left(w_{k}\right) \end{array}$$ (20)

Yuan et al. [50] present a three-term Polak-Ribière-Polyak conjugate gradient algorithm in which the search direction *d*_*k*_ is defined as follows

$$d_{k}=\{\begin{array}{cc} -q_{k} & \text{if}\;k=0\\ -q_{k}+\frac{q_{k}^{T}y_{k-1}d_{k-1}-q_{k}^{T}d_{k-1}y_{k-1}}{\max\{\mu ∥d_{k-1}∥∥y_{k-1}∥,∥q_{k-1}{∥}^{2}\}} & \text{if}\;k\geq 1 \end{array}$$

where *y*_*k*−1_ = *q*_*k*_−*q*_*k*−1_. The derivative-free line search method [48] and the projection-based techniques are used by the algorithm [50], proved to be very suitable for solving large-scale nonlinear equations. The most attractive property of algorithm [50] is the the trust region property of *d*_*k*_.

Motivated by our new modified PRP conjugate gradient formula, proposed in Section 2, we proposed the following modified PRP conjugate gradient formula

$$\begin{array}{c} \beta_{k}^{*}=\frac{\text{min}\left\{\left|q_{k}^{T}\left(q_{k}-q_{k-1}\right)\right|\;,\;u_{3}\left(∥q_{k}{∥}^{2}-\frac{\left\|q_{k}\right\|}{\left\|q_{k-1}\right\|}\left|q_{k}^{T}q_{k-1}\right|\right)\right\}}{u_{4}∥d_{k-1}∥\;∥q_{k}-q_{k-1}∥+∥q_{k-1}{∥}^{2}} \end{array}$$ (21)

and

$$\begin{array}{c} d_{k}=\{\begin{array}{cc} -q_{k}, & \text{if}\;k=1\\ -q_{k}-\beta_{k}^{*}\frac{q_{k}^{T}d_{k-1}}{∥q_{k}{∥}^{2}}q_{k}+\beta_{k}^{*}d_{k-1}, & \text{if}\;k\geq 2 \end{array} \end{array}$$ (22)

**Fig 4:**
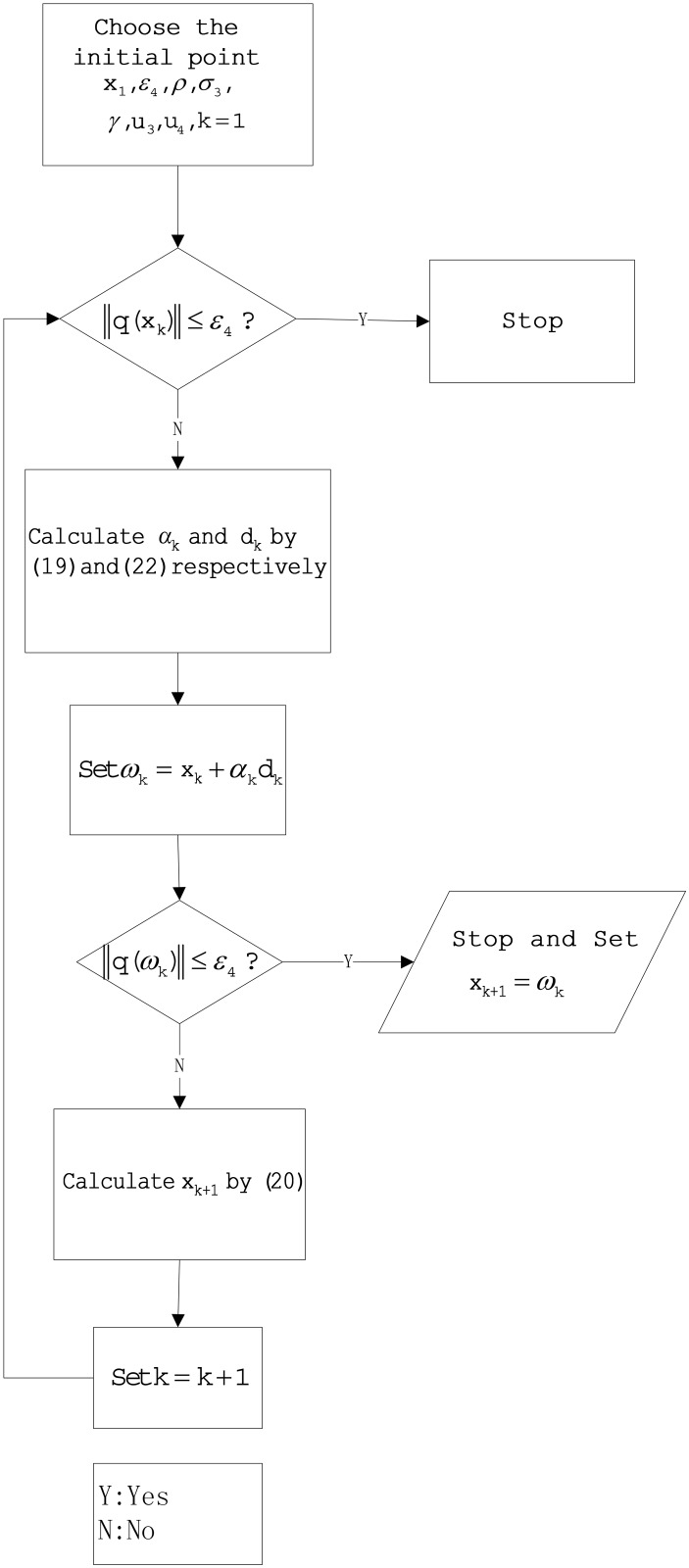
The diagram about Algorithm 5.1.

Where *u*_3_ > 0, *u*_4_ > 0. It is easy to see that $\beta_{k}^{*}\geq 0$, motivated by the above observation and [50]. We present a new algorithm for solving problem Eq (17): it uses our modified PRP conjugate gradient formula Eqs (21) and (22). Here, we list the new algorithm and it’s diagram (Fig 4) as follows.

**Algorithm 5.1**

**Step 1:** Given the initial point *x*_1_ ∈ ℜ^*n*^,*ɛ*_4_ > 0,*ρ* ∈ (0,1), *σ*_3_ > 0, *γ* > 0,*u*_3_ > 0, *u*_4_ > 0, and *k*: = 1.

**Step 2:** If $∥q(x_{k})∥\leq \varepsilon_{4},$ stop; otherwise, go to step 3.

**Step 3:** Compute *d*_*k*_ by Eq (22) and calculate *α*_*k*_ by Eq (19)

**Step 4:** Set the next iterate to be *w*_*k*_ = *x*_*k*_ + *α*_*k*_ *d*_*k*_;

**Step 5:** If $∥q(w_{k})∥\leq \varepsilon_{4}$, stop and set *x*_*k*+1_ = *w*_*k*_; otherwise, calculate *x*_*k*+1_ by Eq (20)

**Step 6:** Set *k*: = *k* + 1; go to step 2.

## Convergence Analysis

When we analyze the global convergence of Algorithm 5.1, we require the following suitable assumptions.

**Assumption 6.1**

1. The solution set of the problem Eq (17) is nonempty.
2. *q*(*x*) is Lipschitz continuous, namely, there exists a constant *E* > 0 such that
  $$∥q(x)-q(y)∥\leq E∥x-y∥,\;\;\forall x,\;y\;\in {ℜ}^{n}.$$

By Assumption 6.1, it is easy to obtain that there exists a positive constant *ζ* that satisfies

$$\begin{array}{c} ∥q(x_{k})∥\leq \zeta \end{array}$$ (23)

**Lemma 0.4** *Let the sequence* {*d*_*k*_} *be generated by* Eq (22) *; then, we can obtain*

$${\begin{array}{c} q_{k}^{T}d_{k}=-\left\|q_{k}\right\| \end{array}}^{2}$$ (24)

*and*

$$\begin{array}{c} ∥q_{k}∥\leq ∥d_{k}∥\leq \left(1+\frac{4u_{3}}{u_{4}}\right)∥q_{k}∥ \end{array}$$ (25)

**Proof** As the proof is similar to Lemma 0.1 and Lemma 0.3 of this paper, we omit it here.

Similar to Lemma 3.1 of [50] and theorem 2.1 of [51], it is easy to obtain the following lemma. Here, we omit this proof and only list it.

**Lemma 0.5** *Suppose that Assumption 6.1 holds and* x* *is a solution of problem* Eq (17) *that satisfies* *g*(x*) = 0. *Let the sequence* {*x*_*k*_} *be obtained by Algorithm 5.1; then, the* {*x*_*k*_} *is a bounded sequence and*

$${\left\|x_{k+1}\;-\;x*\right\|}^{2}\;\leq \;{\left\|x_{k}\;-\;x*\right\|}^{2}\;-\;{\left\|x_{k+1}\;-\;x_{k}\right\|}^{2}$$

*holds. Moreover, either* {*x*_*k*_} *is a infinite sequence and*

$$\sum_{k=1}^{\infty }{∥x_{k+1}-x_{k}∥}^{2}<\infty$$

*or the* {*x*_*k*_} *is a finite sequence and a solution of problem* Eq (17) *is the last iteration*.

**Lemma 0.6** *Suppose that Assumption 6.1 holds, then, an iteration* *x*_*k*+1_ = *x*_*k*_ + *α*_*k*_ *d*_*k*_ *will be generated by Algorithm 5.1 in a finite number of backtracking steps*.

**Proof** We will obtain this conclusion by contradiction: suppose that $∥q_{k}∥\to 0$ does not hold; then, there exists a positive constant *ɛ*_5_ that satisfies

$$\begin{array}{c} ∥q_{k}∥\geq \varepsilon_{5},\;\;\;\forall k\geq 1 \end{array}$$ (26)

suppose that there exist some iterate indexes $k^{{}^{\prime }}$ that do not satisfy the condition Eq (19). We let $\alpha_{k^{{}^{\prime }}}^{\left(c\right)}=\rho^{\left(c\right)}\gamma$ then it can obtain

$$-q{\left(x_{k^{{}^{\prime }}}+\alpha_{k^{{}^{\prime }}}^{\left(c\right)}d_{k^{{}^{\prime }}}\right)}^{T}d_{k^{{}^{\prime }}}<\sigma_{3}\alpha_{k^{{}^{\prime }}}^{\left(c\right)}\;\|q\left(x_{k^{{}^{\prime }}}+\alpha_{k^{{}^{\prime }}}^{\left(c\right)}d_{k^{{}^{\prime }}}\right)\|\;\|d_{k^{{}^{\prime }}}{\|}^{2},\;\forall c\in N^{*}\cup^{}\left\{0\right\}.$$

By Assumption 6.1 (b) and Eq (24), we find

$$\begin{array}{ccc} {\left\|d_{k^{{}^{\prime }}}\right\|}^{2} & = & -q_{k^{{}^{\prime }}}^{T}d_{k^{{}^{\prime }}}\\ & = & {\left[q\left(x_{k^{{}^{\prime }}}+\alpha_{k^{{}^{\prime }}}^{\left(c\right)}d_{k^{{}^{\prime }}}\right)-q\left(x_{k^{{}^{\prime }}}\right)\right]}^{T}d_{k^{{}^{\prime }}}-q{\left(x_{k^{{}^{\prime }}}+\alpha_{k^{{}^{\prime }}}^{\left(c\right)}d_{k^{{}^{\prime }}}\right)}^{T}d_{k^{{}^{\prime }}}\\ & < & \left[E+\sigma_{3}∥q\left(x_{k^{{}^{\prime }}}+\alpha_{k^{{}^{\prime }}}^{\left(c\right)}d_{k^{{}^{\prime }}}\right)∥\right]\alpha_{k^{{}^{\prime }}}^{\left(c\right)}∥d_{k^{{}^{\prime }}}{∥}^{2} \end{array}$$

By Eqs (23) and (25), we can obtain

$$\begin{array}{ccc} ∥q(x_{k^{{}^{\prime }}}+\alpha_{k^{{}^{\prime }}}^{\left(c\right)}d_{k^{{}^{\prime }}})∥ & \leq & ∥q\left(x_{k^{{}^{\prime }}}+\alpha_{k^{{}^{\prime }}}^{\left(c\right)}d_{k^{{}^{\prime }}}\right)-q_{k^{{}^{\prime }}}∥+∥q_{k^{{}^{\prime }}}∥\\ & \leq & E\alpha_{k^{{}^{\prime }}}^{\left(c\right)}∥d_{k^{{}^{\prime }}}∥+\zeta \\ & \leq & E\gamma \zeta (1+\frac{4u_{3}}{u_{4}})+\zeta \end{array}$$

Thus, we obtain

$$\alpha_{k^{{}^{\prime }}}^{\left(c\right)}>\frac{\varepsilon_{5}^{2}u_{4}^{2}}{[E+\sigma_{3}(E\gamma \zeta (1+\frac{4u_{3}}{u_{4}})+\zeta )]{\left(u_{4}+4u_{3}\right)}^{2}\zeta^{2}},\;\forall c\in N^{*}\cup \left\{0\right\}$$

which shows that $\alpha_{k^{{}^{\prime }}}^{\left(c\right)}$ is bounded below. This contradicts the definition of $\alpha_{k^{{}^{\prime }}}^{\left(c\right)}$; so, the lemma holds.

Similar to Theorem 3.1 of [50], we list the following theorem but omit its proof.

**Theorem 0.2** *Let the sequence* {*x*_*k*+1_, *q*_*k*+1_} *and* {*α*_*k*_, *d*_*k*_} *be generated by Algorithm 5.1. Suppose that Assumption 6.1 holds; then, we have*

$$\begin{array}{c} \lim_{k\to \infty }\inf∥q_{k}∥=0. \end{array}$$ (27)

## Numerical results

When the following *d*_*k*_ formula of the famous PRP conjugate gradient method [8, 9]

$$d_{k}=\{\begin{array}{cc} -q_{k} & \text{if}\;k=1\\ -q_{k}+\frac{q_{k}^{T}\left(q_{k}-q_{k-1}\right)}{∥q_{k-1}{∥}^{2}}d_{k-1} & \text{if}\;k\geq 2 \end{array}$$

is used to compute *d*_*k*_ in step 3 of Algorithm 5.1, then it is called PRP algorithm. We test Algorithm 5.1 and the PRP algorithm for some problems in this section. The test environment is MATLAB 7.0 on a Windows 7 system. The initial parameters are given by

$$\sigma_{3}=u_{4}=0.02,\gamma =1,\rho =0.1,u_{3}=1,\varepsilon_{4}=10^{-5}.$$

When the number of iterations is greater than or equal to one thousand and five hundred, the test program will also be stopped. The test results are given in Tables 3 and 4. As we know, when the line search cannot guarantee that *d*_*k*_ satisfies $q_{k}^{T}d_{k}<0$, some uphill search direction may be produced; the line search method possibly fails in this case. In order to prevent this situation, when the search time is greater than or equal to fifteen in the inner cycle of our program, we set *α*_*k*_ that is acceptable. NG, NI stand for the number of gradient evaluations and iterations respectively. Dim denotes the dimension of the testing function, and cputime denotes the cpu time in seconds. GF denotes the evaluation of the final function norm when the program terminates. The test functions all have the following form

$$q\left(x\right)={\left(f_{1}\left(x\right),f_{2}\left(x\right),...,f_{n}\left(x\right)\right)}^{T}$$

the concrete function definitions are given as follows.

**Function 1.** Exponential function 2
  $$\begin{array}{ccc} f_{1}\left(x\right) & = & e^{x_{1}}-1,\\ f_{i}\left(x\right) & = & \frac{i}{10}\left(e^{x_{i}}+x_{i-1}-1\right),\;\;i=2,3,\cdots ,n \end{array}$$
  Initial guess: $x_{0}={\left(\frac{1}{n^{2}},\frac{1}{n^{2}},\cdots ,\frac{1}{n^{2}}\right)}^{T}.$
**Function 2.** Trigonometric function
  $$f_{i}\left(x\right)=2\left(n+i\left(1-\cos\left(x_{i}\right)\right)-\sin\left(x_{i}\right)-\sum_{k=1}^{n}\cos\left(x_{k}\right)\right)\left(2\sin\left(x_{i}\right)-\cos\left(x_{i}\right)\right),\;\;i=1,2,\cdots ,n$$
  Initial guess: $x_{0}={\left(\frac{101}{100n},\frac{101}{100n},\cdots ,\frac{101}{100n}\right)}^{T}.$
**Function 3.** Logarithmic function
  $$f_{i}\left(x\right)=\ln\left(x_{i}+1\right)-\frac{x_{i}}{n},\;\;i=1,2,3,\cdots ,n.$$
  Initial guess: *x*_0_ = (1,1,⋯,1)^*T*^.
**Function 4.** Broyden Tridiagonal function [[52], pp. 471–472]
  $$\begin{array}{ccc} f_{1}\left(x\right) & = & \left(3-0.5x_{1}\right)x_{1}-2x_{2}+1,\\ f_{i}\left(x\right) & = & \left(3-0.5x_{i}\right)x_{i}-x_{i-1}+2x_{i+1}+1,\;\;i=2,3,\cdots ,n-1,\\ f_{n}\left(x\right) & = & \left(3-0.5x_{n}\right)x_{n}-x_{n-1}+1. \end{array}$$
  Initial guess: *x*_0_ = (−1,−1,⋯,−1)^*T*^.
**Function 5.** Strictly convex function 1 [[44], p. 29]
*q*(*x*) is the gradient of $h\left(x\right)=\sum_{i=1}^{n}\left(e^{x_{i}}-x_{i}\right).$
  $$f_{i}\left(x\right)=e^{x_{i}}-1,\;\;i=1,2,3,\cdots ,n$$
  Initial guess: $x_{0}={\left(\frac{1}{n},\frac{2}{n},\cdots ,1\right)}^{T}.$
**Function 6.** Variable dimensioned function
  $$\begin{array}{ccc} f_{i}\left(x\right) & = & x_{i}-1,\;\;i=1,2,3,\cdots ,n-2,\\ f_{n-1}\left(x\right) & = & \sum_{j=1}^{n-2}j\left(x_{j}-1\right),\\ f_{n}\left(x\right) & = & {\left(\sum_{j=1}^{n-2}j\left(x_{j}-1\right)\right)}^{2}. \end{array}$$
  Initial guess: $x_{0}={\left(1-\frac{1}{n},1-\frac{2}{n},\cdots ,0\right)}^{T}.$
**Function 7.** Discrete boundary value problem [53].
  $$\begin{array}{ccc} f_{1}\left(x\right) & = & 2x_{1}+0.5h^{2}{\left(x_{1}+h\right)}^{3}-x_{2},\\ f_{i}\left(x\right) & = & 2x_{i}+0.5h^{2}{\left(x_{i}+hi\right)}^{3}-x_{i-1}+x_{i+1},\;\;i=2,3,\cdots ,n-1\\ f_{n}\left(x\right) & = & 2x_{n}+0.5h^{2}{\left(x_{n}+hn\right)}^{3}-x_{n-1},\\ h & = & \frac{1}{n+1}. \end{array}$$
  Initial guess: *x*_0_ = (*h*(*h*−1), *h*(2*h*−1),⋯,*h*(*nh*−1))^*T*^.
**Function 8.** Troesch problem [54]
  $$\begin{array}{ccc} f_{1}\left(x\right) & = & 2x_{1}+\varrho h^{2}sinh\left(\varrho x_{1}\right)-x_{2}\\ f_{i}\left(x\right) & = & 2x_{i}+\varrho h^{2}sinh\left(\varrho x_{1}\right)-x_{i-1}-x_{i+1},\;\;i=2,3,\cdots ,n-1\\ f_{n}\left(x\right) & = & 2x_{n}+\varrho h^{2}sinh\left(\varrho x_{n}\right)-x_{n-1},\\ h & = & \frac{1}{n+1},\varrho =10. \end{array}$$
  Initial guess: *x*_0_ = (0, 0, ⋯, 0)^*T*^.

**Table 3:** Test results for Algorithm 5.1.

Function	Dim	NI/NG	cputime	GF
1	3000	55/209	2.043613	9.850811e-006
	5000	8/33	0.858005	6.116936e-006
	30000	26/127	100.792246	8.983556e-006
	45000	7/36	62.681202	7.863794e-006
	50000	5/26	56.659563	5.807294e-006
2	3000	43/86	1.076407	8.532827e-006
	5000	42/84	2.745618	8.256326e-006
	30000	38/76	73.039668	8.065468e-006
	45000	37/74	164.284653	8.064230e-006
	50000	36/72	201.288090	9.519786e-006
3	3000	5/6	0.093601	1.009984e-008
	5000	5/6	0.249602	6.263918e-009
	30000	18/33	32.775810	2.472117e-009
	45000	21/39	91.229385	2.840234e-010
	50000	21/39	108.202294	2.661223e-010
4	3000	95/190	2.137214	9.497689e-006
	5000	97/194	5.834437	9.048858e-006
	30000	103/206	194.954450	8.891642e-006
	45000	104/208	446.568463	9.350859e-006
	50000	104/208	549.529123	9.856874e-006
5	3000	64/128	1.497610	9.111464e-006
	5000	65/130	4.102826	9.525878e-006
	30000	70/140	132.117247	8.131796e-006
	45000	70/140	297.868309	9.959279e-006
	50000	71/142	374.964004	8.502923e-006
6	3000	1/2	0.031200	0.000000e+000
	5000	1/2	0.062400	0.000000e+000
	30000	1/2	1.918812	0.000000e+000
	45000	1/2	4.258827	0.000000e+000
	50000	1/2	5.194833	0.000000e+000
7	3000	35/71	0.842405	9.291878e-006
	5000	34/69	2.121614	8.658237e-006
	30000	30/61	58.391174	8.288490e-006
	45000	29/59	135.627269	8.443996e-006
	50000	29/58	153.801386	9.993530e-006
8	3000	0/1	0.015600	0.000000e+000
	5000	0/1	0.046800	0.000000e+000
	30000	0/1	1.326008	0.000000e+000
	45000	0/1	2.917219	0.000000e+000
	50000	0/1	3.510022	0.000000e+000

**Table 4:** Test results for PRP algorithm.

Function	Dim	NI/NG	cputime	GF
1	3000	58/220	2.043613	9.947840e-006
	5000	24/97	2.496016	9.754454e-006
	30000	29/141	109.668703	9.705424e-006
	45000	13/66	118.108357	9.450575e-006
	50000	10/51	112.383120	9.221806e-006
2	3000	48/95	1.138807	8.647042e-006
	5000	46/91	2.932819	9.736889e-006
	30000	41/81	78.733705	9.983531e-006
	45000	40/79	181.709965	9.632281e-006
	50000	40/79	212.832164	9.121412e-006
3	3000	11/12	0.171601	1.012266e-008
	5000	11/12	0.530403	8.539532e-009
	30000	23/38	39.749055	2.574915e-009
	45000	26/44	100.542645	2.931611e-010
	50000	26/44	123.864794	2.838473e-010
4	3000	104/208	2.246414	9.243312e-006
	5000	106/212	6.193240	9.130520e-006
	30000	113/226	219.821009	8.747379e-006
	45000	114/228	487.908728	9.368026e-006
	50000	114/228	611.976323	9.874918e-006
5	3000	35/53	0.561604	2.164559e-006
	5000	35/53	1.716011	1.291210e-006
	30000	35/53	55.926358	1.336971e-006
	45000	33/49	116.361146	2.109293e-006
	50000	33/49	147.452145	2.225071e-006
6	3000	1/2	0.031200	0.000000e+000
	5000	1/2	0.062400	0.000000e+000
	30000	1/2	1.965613	0.000000e+000
	45000	1/2	4.290028	0.000000e+000
	50000	1/2	5.257234	0.000000e+000
7	3000	40/80	0.904806	9.908999e-006
	5000	39/78	2.386815	9.198351e-006
	30000	34/68	66.440826	9.515010e-006
	45000	33/66	140.026498	9.366998e-006
	50000	33/66	173.597913	8.886013e-006
8	3000	0/1	0.015600	0.000000e+000
	5000	0/1	0.031200	0.000000e+000
	30000	0/1	1.279208	0.000000e+000
	45000	0/1	2.808018	0.000000e+000
	50000	0/1	3.432022	0.000000e+000

By Tables 3 and 4, we see that Algorithm 5.1 and the PRP algorithm are effective for solving the above eight problems.

We use the tool of Dolan and Morè [35] to analyze the numerical performance of the two algorithms when NI, NG and cputime are considered, for which we generate three figures.

**Fig 5:**
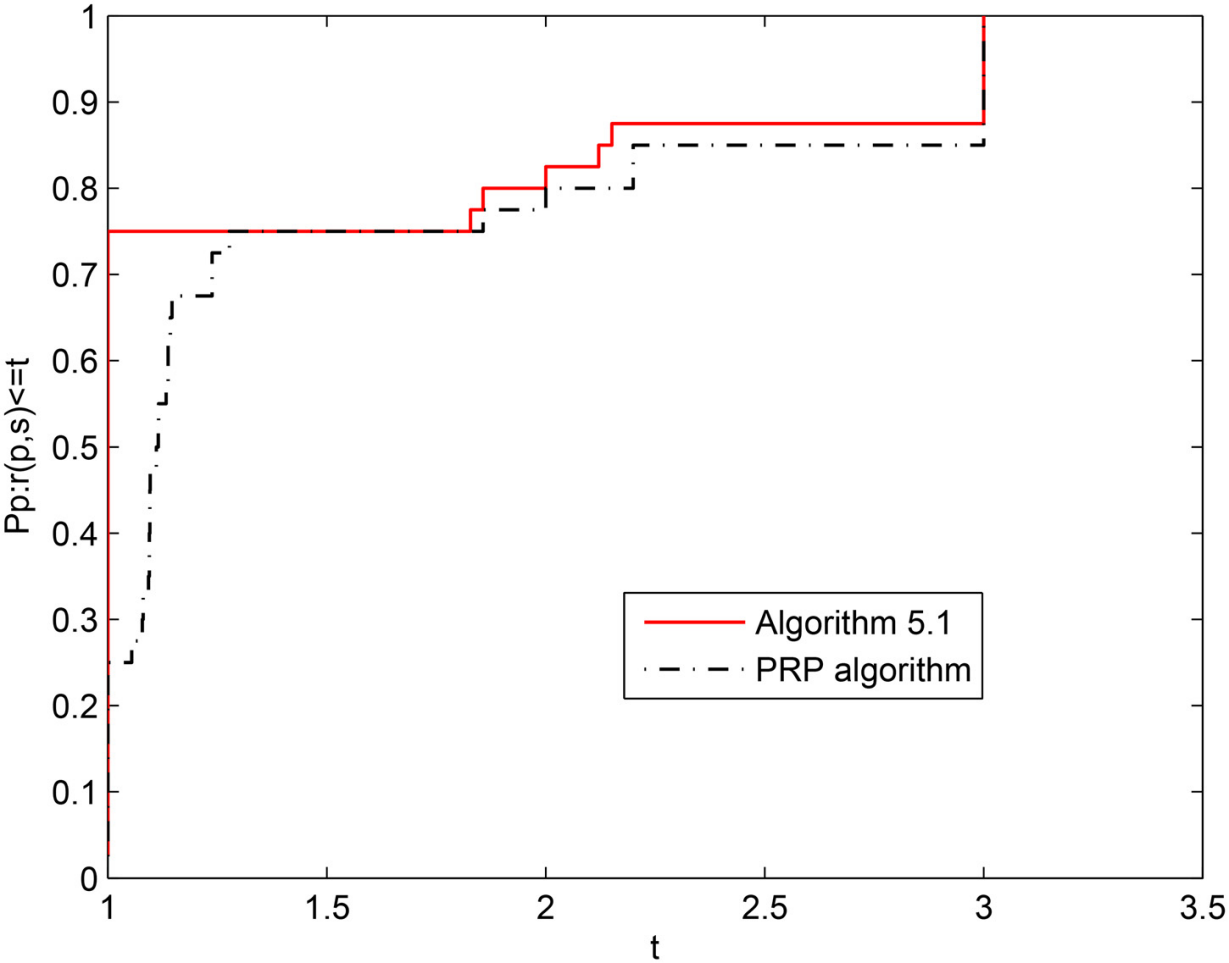
Performance profiles of the two algorithms (NI).

**Fig 6:**
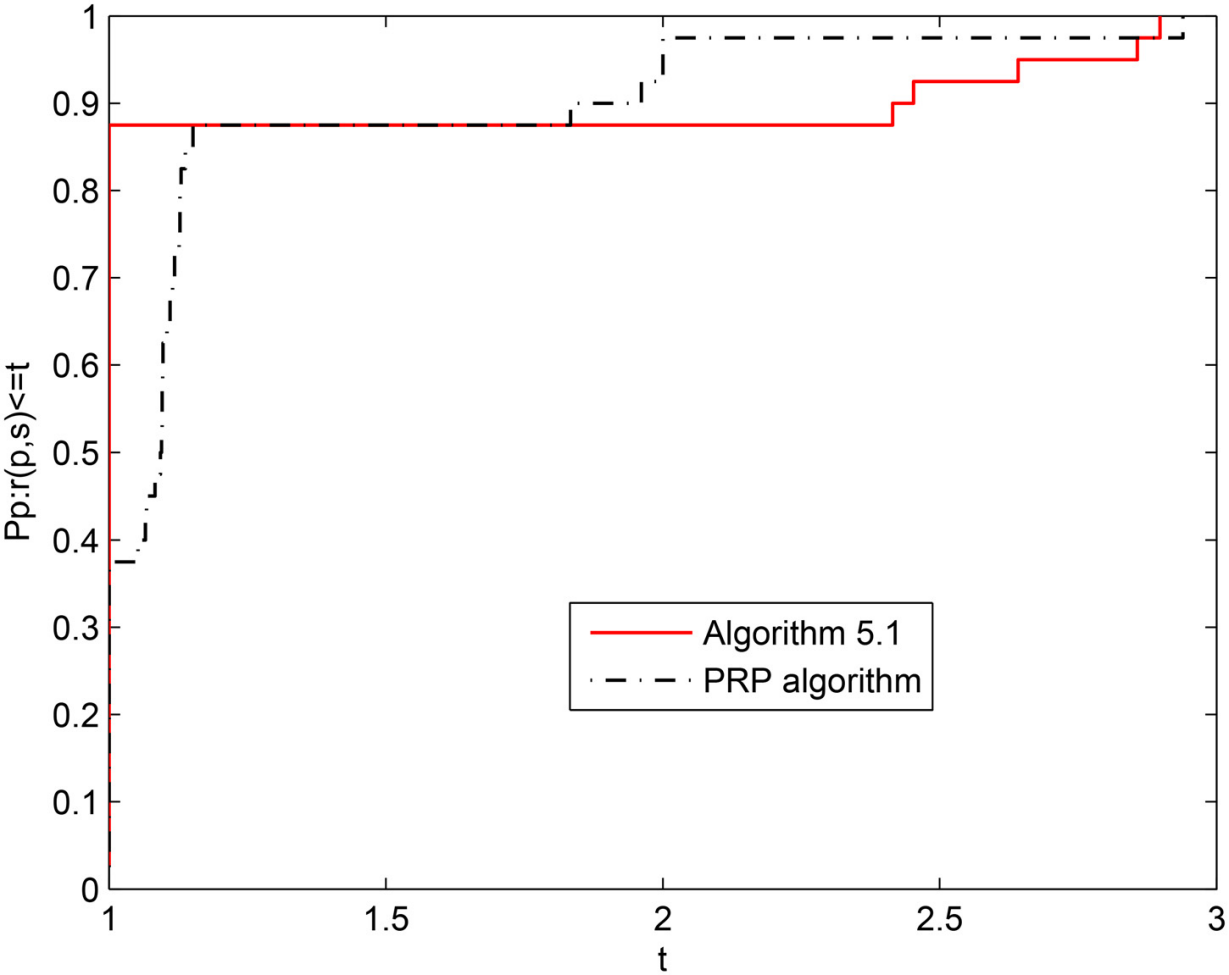
Performance profiles of the two algorithms (NG).

**Fig 7:**
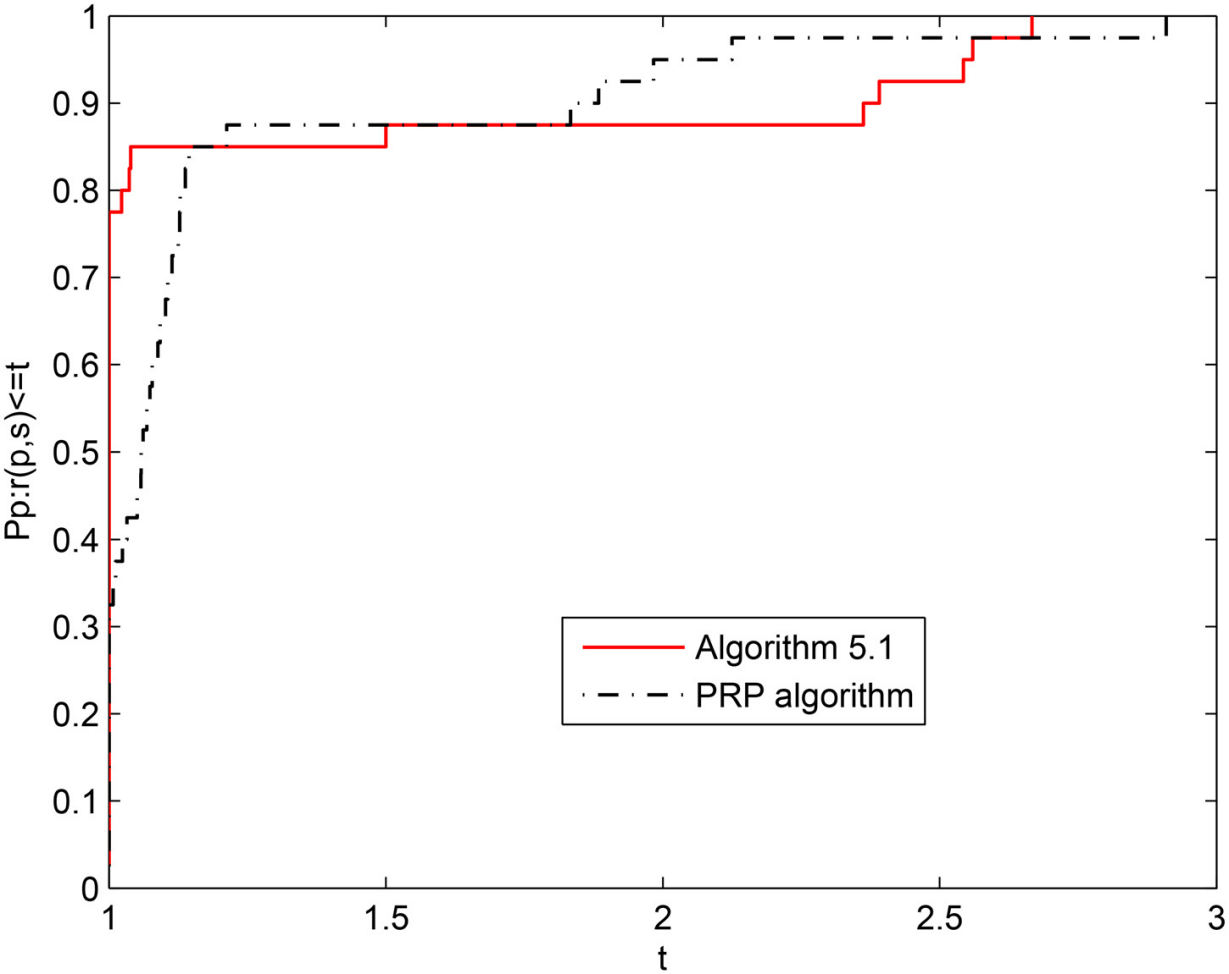
Performance profiles of the two algorithms (cputime).

Fig 5 shows that the numerical performance of Algorithm 5.1 is slightly better than that of the PRP algorithm when NI is considered. It is easy to see that the numerical performance of Algorithm 5.1 is better than that of the PRP algorithm from Figs 6 and 7 because the PRP algorithm requires a bigger horizontal axis when the problems are completely solved.

From the above two tables and three figures, we see that Algorithm 5.1 is effective and competitive for solving large-scale nonlinear equations.

## Conclusion

(i) This paper provides the first new algorithm based on the first modified PRP conjugate gradient method in Sections 1–4. The *β*_*k*_ formula of the method includes the gradient value and function value. The global convergence of the algorithm is established under some suitable conditions. The trust region property and sufficient descent property of the method have been proved without the use of any line search method. For some test functions, the numerical results indicate that the first algorithm is effective and competitive for solving unconstrained optimization problems.

(ii) The second new algorithm based on the second modified PRP conjugate gradient method is presented in Sections 5-7. The new algorithm has global convergence under suitable conditions. The trust region property and the sufficient descent property of the method are proved without the use of any line search method. The numerical results of some tests function are demonstrated. The numerical results show that the second algorithm is very effective for solving large-scale nonlinear equations.
